# The Home Environment Interview and associations with energy balance behaviours and body weight in school-aged children – a feasibility, reliability, and validity study

**DOI:** 10.1186/s12966-021-01235-3

**Published:** 2021-12-23

**Authors:** Alice R. Kininmonth, Stephanie Schrempft, Andrea Smith, Louise Dye, Clare Lawton, Abigail Fisher, Clare Llewellyn, Alison Fildes

**Affiliations:** 1 School of Psychology, University of Leeds, Leeds, LS2 9JT UK; 2 Unit of Population Epidemiology, Division of Primary Care, Geneva University Hospitals, Geneva, Switzerland; 3 MRC Epidemiology Unit, University of Cambridge, Cambridge, UK; 4 University College London, London, UK

**Keywords:** Home environment interview, Childhood, Food, Physical activity, Sedentary behaviour, Media

## Abstract

**Background:**

The home environment is thought to influence children’s weight trajectories. However, few studies utilise composite measures of the home environment to examine associations with energy balance behaviours and weight. The present study aimed to adapt and update a comprehensive measure of the obesogenic home environment previously developed for pre-schoolers, and explore associations with school-aged children’s energy balance behaviours and weight.

**Methods:**

Families from the Gemini cohort (*n* = 149) completed the Home Environment Interview (HEI) via telephone when their children were 12 years old. The HEI comprises four composite scores: one for each domain (food, activity and media) of the environment, as well as a score for the overall obesogenic home environment. The primary caregiver also reported each child’s height and weight (using standard scales and height charts), diet, physical activity and sedentary screen-based behaviours. A test-retest sample (*n* = 20) of caregivers completed the HEI a second time, 7–14 days after the initial interview, to establish test-retest reliability.

**Results:**

Children (*n* = 298) living in ‘higher-risk’ home environments (a 1 unit increase in the HEI obesogenic risk score) were less likely to consume fruits (OR; 95% CI = 0.40; 0.26–0.61, *p* < 0.001), and vegetables (0.30; 0.18–0.52, *p* < 0.001), and more likely to consume energy-dense snack foods (1.71; 1.08–2.69, *p* = 0.022), convenience foods (2.58; 1.64–4.05, *p* < 0.001), and fast foods (3.09; 1.90–5.04, *p* < 0.001). Children living in more obesogenic home environments also engaged in more screen-time (*β (SE)* = 4.55 (0.78), *p* < 0.001), spent more time playing video games (*β (SE)* = 1.56 (0.43), *p* < 0.001), and were less physically active (OR; 95% CI = 0.57; 0.40–0.80, *p* < 0.01). Additionally, there was a positive association between higher-risk overall home environment composite score and higher BMI-SDS *(β (SE) = 0.23 (0.09), p < 0.01)*. This finding was mirrored for the home media composite (*β (SE) = 0.12* (0.03), *p* < 0.001). The individual home food and activity composite scores were not associated with BMI-SDS.

**Conclusion:**

Findings reveal associations between the overall obesogenic home environment and dietary intake, activity levels and screen-based sedentary behaviours, as well as BMI in 12 year olds. These findings suggest that the home environment, and in particular the home media environment, may be an important target for obesity prevention strategies.

**Supplementary Information:**

The online version contains supplementary material available at 10.1186/s12966-021-01235-3.

## Background

The home environment has been shown to play an influential role in shaping children’s food intake [1, 2], physical activity levels, and screen-based sedentary behaviours [3–5]. Various socio-ecological models have been developed to conceptualise how different aspects of the home environment may influence children’s growth and development [6–8]. The ‘obesogenic’ home environment can be categorised into three domains: food, physical activity and media. Each domain consists of physical (e.g. availability and access) and social factors (e.g. caregiver modelling, rules and limit setting) that have been associated with children’s food intake [1], physical activity, and sedentary behaviours [3, 9, 10]. Evidence highlights that these aspects of the home environment may predict children’s weight trajectories [6, 11], and thus may be important for obesity prevention and treatment strategies [2, 12, 13].

Despite the importance of the home environment, there are few measures that comprehensively assess both physical and social aspects. A recent systematic review highlighted that existing measures are limited, with most focussing on individual aspects of a single domain (e.g. availability of fruits and vegetables in the home, access to TV in bedroom, etc.), rather than assessing the overall obesogenic home environment [11]. As individual aspects of the home environment are likely to have a limited influence on children’s weight-related outcomes, comprehensive measures are required to better understand how, and to what extent the home environment relates to children’s energy balance behaviours and subsequently weight. Additionally, many existing measures of the home environment lack appropriate evaluation, or reporting of, the psychometric properties (e.g. validity and reliability) of the measurement tool [11, 14].

One of the few comprehensive measures of obesogenic risk in the home environment was developed by Schrempft et al. [15] for use in pre-school children. The Home Environment Interview (HEI) comprises four composite scores; one for each individual domain (food, activity and media) of the environment, as well as a score for the overall obesogenic home environment. This measure was found to be reliable over a 2 week period, and showed good validity when compared against data from objective wearable recording devices [16]. Findings indicated cross-sectional associations between the home environment composite score and food intake, physical activity levels and sedentary behaviours in pre-school children, but no relationships were found with child weight [15]. The home environment composite scores were also associated with heritability of BMI [17], with higher heritability of BMI observed among children living in higher-risk home environments compared to lower-risk home environment (86% vs 39%). This suggests the home environment moderates the extent to which the genetic influence on BMI is expressed, and indicates the family home may offer some protection for children who are genetically susceptible to obesity. It is possible that 4 years of age is too young for the relationship between the obesogenic quality of the home environment and body weight to be fully expressed or observed, but these relationships may manifest later in childhood, following longer exposure. The aim of the present study was: (i) to update and adapt the home environment interview, for use in school-aged children, and (ii) to examine associations between the home environment composites and energy balance behaviours and BMI-SDS in school-aged children.

## Methods

### Instrument development: home environment interview

The original HEI assesses both physical (e.g. availability and accessibility of foods) and social (e.g. parental modelling and support of healthy eating) aspects of the food, physical activity and media domains within the home environment and was validated in pre-school children (Schrempft et al., 2015). This study updated and validated the revised HEI for use in older children in three phases.

In the first phase, the original HEI was circulated to a panel of six experts in the field of childhood obesity to gather input and achieve consensus about the relevance of existing items, alongside suggestions for additional items. The expert consultation highlighted the need to widen the scope of questions about the media environment, to reflect technological advances (e.g. increases in amount and types of devices available) and changes in how children use and interact with media since the development of the original HEI in 2012. Peer-reviewed published studies examining the home media environment were identified and the measures used were reviewed. The largest national survey of media and electronic devices in the UK (The Ofcom report) was also reviewed [18, 19]. Where possible, these resources were used to refine and modify the wording of the existing HEI questions, and to add additional questions. The instrument was iteratively refined based on feedback from the panel of aforementioned experts.

In the second phase, one-to-one cognitive interviews were conducted with a sample of parents (*n* = 14) of children aged 11–13 years old living in the U.K. Participants were recruited via social advertisements and word-of-mouth. Cognitive interviewing techniques were utilised to ascertain parents’ comprehension of items and response options (e.g. clarity of interpretation and understanding), and the acceptability and relevance of items. Modifications were made to the HEI based on feedback gained at this stage. Overall, cognitive interviews revealed good acceptability, comprehension and face validity.

In the third and final phase, a further panel of experts in the field of childhood obesity research were consulted using a Delphi method [20]. Expert opinion was sought between March and June 2020 to gain consensus about the constructs to include in the composite scores. Fifty-four experts from the US (*n* = 24), Europe (*n* = 20) and Australia/New Zealand (*n* = 10) were contacted via email and invited to complete an online questionnaire anonymously. Twenty-one (39%) experts completed the survey and were presented with each of the proposed HEI variables. Experts were asked to indicate whether each of the items were associated with an increased or decreased risk of weight gain in childhood (response options; ‘probably/definitely associated with decreased risk for weight gain’, ‘not sure’, or ‘probably/definitely associated with increased risk for weight gain’). There was also a free text box for experts to provide additional comments. The results of the survey are provided in Table S1.

### Construct validity: HEI instrument administration

The final version of the HEI was administered as a computer-assisted telephone interview by a trained researcher using the secure, web-based software platform REDCap (Research Electronic Data Capture) [21, 22]. Study data were collected and managed using REDCap electronic data capture tools hosted at University College London (UCL). Primary caregivers were asked to complete the interview at home in one sitting and were prompted to check the foods and beverages in their home, to ensure accurate responding. The interview took around 45 min to complete. Parental feeding practices were assessed using validated questionnaires, which were completed online by caregivers in the weeks prior to completing the HEI [23–26].

### Construct validity sample

Participants were from the Gemini study, a longitudinal birth cohort of families with twins born in England and Wales between March and December 2007. A total of 2402 families with monozygotic (identical) and dizygotic (non-identical) twins consented to take part. Additional details are provided elsewhere [27]. Families were previously invited to take part in a home environment interview (HEI) when the children were on average 4.2 years old (SD = 0.4) (Schrempft et al., 2015). Only families who had taken part in the HEI at age 4 (*n* = 1113), and those who completed parental feeding practices questionnaires in the month prior (*n* = 219), were invited to take part in the present study.

### Test-retest reliability sample

A convenience sample of participants were invited to take part in the HEI a second time, 7–14 days after initial interview (mean ± SD days = 10.6 ± 3.02), to examine test-retest reliability of the measure. Due to the Covid-19 coronavirus pandemic, data collection in the Gemini cohort was halted early and consequently the remainder of the test-retest sample was recruited from the general population when stay at home restrictions had eased in summer 2021. As such, information about birth weight, gestational age, BMI-SDS or maternal BMI for the test-retest sample could not be collected. A total of 20 caregivers took part in this portion of the data collection, whose children were aged on average 12.4 (±0.74) years old at the time of reporting.

### Creating the composite score of obesogenic home environment

A Delphi method was used to gain expert consensus about the relevant constructs for inclusion in the composite scores. A variable was included in the composite if the majority (60% or more) of experts (*n* = 21) identified it as being associated with increased or decreased risk for weight gain (Table S1).

Constructs identified as being associated with a decreased risk for childhood weight gain were reverse scored so that a higher total score on each composite would reflect ‘higher-risk’ for weight gain. The HEI contains continuous, categorical, and ordinal variables. Therefore, to ensure all variables were on a common scale each variable was standardised using z-scores. Before standardising the food and beverage availability variables (vegetables, fruit, salty snacks, sweet snacks, confectionery and sugar-sweetened beverages), linear regression analyses were conducted to examine relationships with ‘typical’ availability (more than usual, less than usual or about the same), and the number of days since the participant last shopped for food/drink. In each regression model, the particular food/beverage availability was the dependent variable (DV) and how typical the reported availability was and the days since last shopped were the independent variables (IVs). If only one of the IVs was significantly associated with food/drink availability, the model was re-run to include just the significant variable and the standardised residuals for the model were used in the composite [15]. This method was used to account for how typical the food in the home was at the time of data collection compared to ‘usual’. To create the standardised energy-dense snack availability variable, the standardised residuals for salty snack, sweet snack and confectionery availability were summed. The variable was then standardised again to have a mean of 0 and a standard deviation of 1.

Finally, the standardised variables (Z-scores) were summed to create three composites: the home food environment (21 variables), the home physical activity environment (6 variables), and the home media environment (5 variables). The food, PA and media composites were then summed to create an overall home environment composite, dividing by the number of variables per composite so that each composite contributed equally to the overall score (food composite/21 + activity composite/6 + media composite/5). Higher scores on each composite scale reflect ‘higher-risk’ environments.

The final list of constructs included in the composite, with descriptive statistics, are detailed in Table 1. This updated composite score was similar in structure to the original HEI composite score [15], however, notable changes were made to constructs included in the home media domain. Full modifications are shown in Table S2.

**Table 1:** Descriptive statistics for the variables included in the HEI composite scores (*n* = 149 families; *n* = 298 children), mean (SD) for continuous variables and percentage (N) for categorical variables

Home food environment	Mean (SD) or % (N)
***Availability***	
Number of fruit types^a^	9.65 (4.25)
Number of vegetable types^a^	13.58 (4.63)
Number of energy-dense snack types	6.96 (3.22)
Number of sugar-sweetened drinks	1.44 (1.05)
***Accessibility (visibility)***	
Fruit on display^a^	95.3 (284)
Vegetables ready-to-eat^a^	43 (128)
Energy-dense snacks on display	4.0 (12)
Sugar-sweetened drinks on display	6.0 (18)
***Accessibility (child can help him/herself)***	
Fruit^a^	92.6 (276)
Vegetables^a^	94.6 (282)
Energy-dense snacks	55.4 (165)
Sugar-sweetened drinks	41.6 (124)
***Parental feeding practices***	
Emotional feeding^b^	1.45 (0.47)
Instrumental feeding^b^	1.81 (0.53)
Encouragement^a, b^	2.28 (0.59)
Modelling^a, b^	3.65 (0.68)
Monitoring^a, b^	2.44 (0.98)
Covert restriction^a, b^	3.23 (0.89)
Restriction^a, b^	3.52 (1.16)
Family meal frequency at the table (days per /week)	3.43 (2.18)
Frequency child eats while watching TV and/or using a device (days per /week)	1.66 (1.09)
**Home activity environment**	
Garden/outdoor space^a^	98.7 (294)
Garden play equipment^a, d^	65.8 (196)
Allowed to be physically active indoors^a, d, e^	4.30 (1.07)
Allowed to be physically active outdoors^a, d, e^	4.76 (0.56)
Parental modelling of physical activity^a^	3.97 (0.96)
Parental support of physical activity^a^	3.53 (0.77)
**Home media environment**	
Number of media equipment items in home	15.48 (4.20)
Number of media equipment in child’s bedroom	1.70 (1.37)
Caregiver rules around use of media equipment^a,f^	2.38 (0.78)
Maternal time engaged in screen-based viewing (hours/week)	14.26 (8.55)
Partner time engaged in screen-based viewing (hours/week)	14.94 (9.61)

^a^Variables identified as being associated with decreased risk for weight gain were reverse scored

^b^Measured using a five-point Likert scale (1 = never, 5 = always)

^c^Measured using a seven-point Likert scale (1 = not at all, 7 = strictly)

^d^*n* = 294 as four children did not have access to a garden or outdoor space

^e^Measured using a five-point Likert scale (1 = never; 5 = all the time)

^f^(0 = no rules, 1 = rules around one device, 2 = rules around two devices, 3 = rules around 3 or more devices)

### Energy balance behaviours

#### Dietary intake

Parents were asked to report how often their children consumed fruit (excluding fruit juice), vegetables, energy-dense snacks (e.g. crisps and chocolate), sugar-sweetened drinks, artificially-sweetened drinks, milk, fruit juice and smoothies. Responses were recorded on an eight-point scale (1 = never or less than once a month, 2 = 1–3 times a month, 3 = once a week, 4 = 2–4 times a week, 5 = 5–6 times a week, 6 = once a day, 7 = 2–3 times per day, 8 = four or more times per day). The questions were based on those used in brief dietary assessment methods, such as the Dietary Instrument for Nutrition (DINE), which has been validated against 4-day diet diaries [28]. In accordance with the 5-a-day UK dietary recommendation, fruit and vegetable consumption was categorised so that the higher consumption group represented consuming two or more portions a day. Energy-dense snack, sugar-sweetened and artificially sweetened beverage consumption were collapsed so the highest consumption group represented consuming once or more per day.

#### Physical activity levels

Physical activity levels were assessed using the item ‘Compared to other children of the same age and sex, how physically active is your child?’ with a five-point response scale (1 = much less active, 2 = somewhat less active, 3 = average, 4 = somewhat more active, 5 = much more active); which has been shown to be associated with objectively measured physical activity at age 11 (*β (SE) = 60.5 (17.0), p < 0.01)* [29]. For ease of interpretation, physical activity level was categorised so that the active group included those who were more active (response 4; somewhat more active and 5; much more active) than other children of the same age and sex; the comparison group were less active (response 1; much less active, 2; somewhat less active; or 3; about average).

#### Sedentary behaviours

Parents were asked to report children’s use of electronic devices to watch TV or other online media using the item ‘On average, how long does your child watch TV programmes, movies, or online media (e.g. Netflix, Amazon Prime, YouTube videos) on an electronic device (e.g. desktop computer/laptop/tablet computer) on a typical weekday (Monday to Friday), at this time of year?’. Parents were also asked to report children’s video game use using the item ‘On average, how long does your child spend playing video games on a typical weekday (Monday to Friday), at this time of year? This includes on a handheld device, games console or computer/laptop.’ There are no specific guidelines for duration of screen-time and video game use in this age group [30], therefore media use was kept as a continuous variable.

#### Socioeconomic status

Parents provided information about multiple indicators of SES, including: highest maternal educational qualification; current occupation (both parents); total annual household income; postcode; home ownership status; number of bedrooms in the home; and number of cars. Principal component analysis was used to create the SES composite score, which incorporated these seven indicators of SES. Higher composite scores reflect higher SES. Full details of the SES composite are described elsewhere [31].

#### Anthropometric measurements at 12 years

Electronic weighing scales were sent to all Gemini families when the children were 2 years old and updated height charts were sent when the children were 10 years old to collect measurements at 3-month intervals. At the time of the HEI, parents were also asked to provide their child’s height and weight measurements. Child date of birth (used to calculate age at the time of the interview), sex and gestational age were parent reported at baseline. Standard deviation scores (SDS) for child BMI (BMI-SDS) were calculated using the UK90 British growth reference data [32], adjusting for age at the time of measurement, sex, and gestational age.

### Statistical analysis

All analyses were performed using SPSS version 26.0 [33], with a *p*-value < 0.05 considered statistically significant.

Single measure intraclass correlation coefficients (ICC) with 95% confidence intervals (95% CI) were used to assess test-retest reliability of each home environment composite score. ICC values were categorised as < 0.40 = poor, 0.40–0.75 = fair to good agreement, > 0.75 = excellent [34].

For categorical outcomes, Complex Samples Logistic Regression was used to examine associations between domain-specific home environment composites and corresponding diet, physical activity and sedentary behaviours of each individual child. For continuous outcomes, Complex Samples General Linear Models were used to examine associations between domain specific home environment composites and corresponding sedentary behaviours. Analyses were adjusted to account for clustering within families (complex samples analyses), sex of child and the child’s age at time of home environment interview.

## Results

### Sample characteristics

In total, 219 families were invited to take part and 149 families (68.0%) with 298 children participated in the current study. There were no significant differences in baseline characteristics (i.e. age of mother, maternal BMI, SES, gestational age) between those invited to take part and the final sample. The study sample comprised families with data on all variables included in the analysis. Primary caregivers completed the HEI by telephone when the twins were on average 12.51 years old (SD = 0.22). Of responding caregivers, 98.7% (*n* = 147) were the child’s mother and 1.3% (*n* = 2) were the father. The mean (±SD) duration of interviews was 44.65 (±10.73) minutes. Characteristics of the sample are shown in Table 2.

**Table 2:** Characteristics of families in the HEI sample at age 12 and test-retest reliability sample, mean (SD) for continuous variables and percentage (N) for categorical variables

	HEI at age 12 (***n*** = 149 families, 298 children)	Test-retest sample (***n*** = 20 families)
	Mean (SD) or % (n)	Mean (SD) or % (n)
Age of child at HEI (years)	12.51 (0.22)	12.40 (0.74)
Gestation (weeks)	36.07 (2.6)	–
Birth weight SDS	−0.57 (0.96)	–
BMI SDS at age 12	−0.06 (1.14)	–
**Sex of child**		
Male	48.7 (145)	55.0 (11)
Female	51.3 (106)	45.0 (9)
**Zygosity**		
MZ	28.9 (43)	–
DZ	70.5 (105)	–
**Maternal age at twin’s birth** (years)	35.1 (4.22)	32.8 (5.94)
**Maternal BMI at baseline**^a^	24.26 (4.22)	–
**SES**^b^**composite at baseline**	5.03 (1.01)	–
**SES**^b^**composite at HEI**	5.15 (1.03)	4.94 (0.97)
**Maternal ethnicity**		
White	94.6 (141)	100 (20)
Non-white	5.4 (8)	0 (0)
**Marital status**		
Married or cohabiting	94 (140)	80 (16)
Separated or divorced	4 (6)	10 (2)
Single	2 (3)	10 (2)
**Child’s dietary intake**		
*Fruit consumption*		
≥ twice a day	58.1 (173)	60.0 (12)
< twice a day	41.9 (125)	40.0 (8)
*Vegetable consumption*		
≥ twice a day	80.2 (239)	65.0 (13)
< twice a day	19.8 (59)	35.0 (7)
*Energy-dense snack consumption*		
≥ once a day	75.2 (224)	80.0 (16)
< once a day	24.8 (74)	20 (4)
*Fast food consumption*		
≥ once a week	19.8 (59)	20.0 (4)
Never or less than once a week	80.2 (239)	80.0 (16)
*Convenience food*		
≥ twice per week	35.6 (106)	35.0 (7)
< twice per week	64.4 (192)	65.0 (13)
*Sugar-sweetened drink consumption*		
≥ once a day	8.4 (25)	0.0 (0)
< once a day	91.6 (273)	100.0 (20)
*Artificially-sweetened drink consumption*		
≥ once a day	67.4 (201)	65.0 (13)
< once a day	32.6 (97)	35.0 (7)
*Fruit juice & smoothie consumption*		
≥ once a day	41.9 (125)	45.0 (9)
< once a day	58.1 (173)	55.0 (11)
*Milk consumption*		
≥ twice a day	71.4 (212)	40.0 (8)
< twice a day	28.6 (85)	60.0 (12)
*Physical activity level*^c^		
Somewhat or much more active	59.4 (177)	55.0 (11)
About average or less active	40.6 (121)	45.0 (9)
*Sedentary behaviours*		
TV viewing and online media use (hours/week)	16.73 (9.70)	13.37 (7.52)
Video game use (hours/week)	6.91 (6.82)	6.13 (8.58)
**Home environment composites**	**Range**	
Home food environment composite	−13.67-23.15	–
Home PA environment composite	−4.54-15.45	–
Home media environment composite	−5.45-9.31	–
Overall home environment composite	−2.17-3.02	–

^a^Data were missing for 0.7% (*n* = 1) families

^b^The SES composite score is a weighted score which takes into account the following indicators of SES: gross annual household income (before tax deductions), index of multiple deprivation (IMD), maternal education, home ownership status, household National Statistics Socioeconomic classification (NS-SEC) based on the household representative person, number of bedrooms and number of cars [31]

^c^Compared to other children of the same age and sex

When examining differences based on SES, lower SES was associated with ‘higher-risk’ overall home environments (*r* = −.26, *p* = .002), as well as ‘higher-risk’ home activity environments (r = −.21, *p* = .01) and ‘higher-risk’ home media environments (r = −.20, *p* = .014). No association was observed between SES and the home food environment (r = −.05, *p* = .529).

### Test-retest reliability

Test-retest reliability (ICC; 95% CI) of the home environment composite scores over a mean period of 10.6 (±3.02) days were excellent for food (0.77; 0.52–0.90), media (0.83; 0.61–0.93) and the overall score (0.76; 0.49–0.90), and were good for activity (0.62; 0.27–0.83).

### Construct validity

The ranges (for the standardised scores) on each home environment composite indicated that there was considerable variation between households: food (− 13.67–23.15), physical activity (− 4.54–15.45), media (− 5.45–9.31) and overall (− 2.17–3.02). Associations between the composites were low for food and activity (*r* = .21, *p* < .001), and moderate for media and food (*r* = .37, *p* < .001), and for the activity and media (*r* = .05, *p* = .579).

As shown in Table 3, for each 1 unit increase in obesogenic risk in the home food environment children were 11% less likely to consume fruits at least twice per day (OR; 95% CI = 0.89; 0.84–.96; *p* < .001) and 12% less likely to consume vegetables at least twice per day (OR: 0.88; OR: 0.83–0.93; *p* < .001). On the other hand, for each 1 unit increase in obesogenic risk in the home food environment children were 13% more likely to consume energy-dense snacks at least once per day (OR: 1.13; 1.05–1.21; *p* < .001), 15% more likely to consume fast foods at least once per week (OR: 1.15; 1.07–1.23; *p* < .001) and 11% more likely to consume convenience foods at least twice per week (OR: 1.11; 1.05–1.17, *p* = .001). There were no significant associations between the home food environment and children’s consumption of sugar-sweetened beverages, artificially sweetened beverages, fruit juice or milk (ns; see Table 3).

**Table 3:** Complex samples logistic regression and CSGLM^a^: associations between food, physical activity and media home environments and corresponding diet, physical activity and screen-based sedentary behaviours (*n* = 298)

	Home food environment			
**Outcome variables**	**N (%)**	**OR (95%CI)**^**1**^		***P value***
**Dietary intake behaviours**				
Fruit (≥twice per day)	173 (58.1%)	**0.89 (0.84–0.96)**		**<.001**
Vegetables (≥twice per day)	239 (80.2%)	**0.88 (0.83–0.93)**		**<.001**
Energy-dense snacks (≥once per day)	224 (75.2%)	**1.13 (1.05–1.21)**		**<.001**
Fast food intake (≥once per week)	59 (19.8%)	**1.15 (1.07–1.23)**		**<.001**
Convenience food (≥twice per week)	106 (35.6%)	**1.11 (1.05–1.17)**		**.001**
Sugar Sweetened Beverages (≥once per day)	25 (8.4%)	1.03 (0.97–1.10)		.334
Artificially-sweetened beverages (≥once per day)	97 (32.6%)	1.05 (0.99–1.10)		.084
Fruit juice (≥once per day)	125 (41.9%)	0.98 (0.94–1.03)		.508
Milk (≥twice per day)	85 (28.6%)	1.00 (0.95–1.06)		.995
**Activity behaviours**	**Home physical activity environment**			
Physical activity (more active)	177 (59.4%)	0.89 (0.78–1.03)		.130
**Screen-based sedentary behaviours**^b^	**Home media environment**			
	**Mean (SD)**	**B (**±**SE)**	**R**^**2**^	***P value***
TV viewing and online media (hours/ week)	16.73 (9.70)	**1.85 (**±**0.24)**	**.276**	**<.001**
Video games (hours/ week)	6.91 (6.82)	**0.61 (**±**0.14)**	**.344**	**<.001**

*OR* Odds Ratio, 95% CI 95% confidence interval

^a^Adjusting for clustering within families (complex samples analyses), the child’s age at time of home environment interview, child sex

^b^Screen-based sedentary behaviours were treated as a continuous variable as there are no specific guidelines for duration of screen-time and video game use in this age group (Hill et al., 2016)

No association was observed between home physical activity environments and children’s physical activity levels. However, for each 1 unit increase in obesogenic risk in the media environments children were 11% more likely to be less physically active than other children (OR; 95% CI = 0.89; 0.80–0.99, *p* = .037). Children living in ‘higher-risk’ media environments also had higher overall screen time (TV viewing and online media: *β (SE)* = 1.85 (.24), *p* < .001) and higher video game use (*β (SE)* = 0.61 (0.14), *p* < .001), such that children’s overall screen-time was 1.87 units (hours/week) higher and video game use was 0.61 units (hours/week) higher for each 1 unit increase in obesogenic risk in the home media environment.

Similar findings were observed for the overall home environment (Table 4), each 1 unit increase in obesogenic risk in the home environment was associated with children being 60% less likely to consume fruit at least twice per day (OR; 95% CI = 0.40; 0.26–0.61, *p* < .001), 70% less likely to consume vegetables at least twice per day (OR: 0.30; 0.18–0.52, *p* < .001) and 71% more likely to consume energy-dense snacks at least once per day (OR: 1.71; 1.08–2.69, *p* = 0.022), 3 times more likely to consume fast food at least once per week (OR: 3.09; 1.90–5.04, *p* < 0.001), and 2.6 times more likely to consume convenience foods at least twice per week (OR: 2.58; 1.64–4.05, *p* < 0.001).

**Table 4:** Complex samples logistic regression and CSGLM^a^: associations between overall home environment composite and corresponding diet, physical activity and screen-based sedentary behaviours (*n* = 298)

Dietary intake behaviours	Home environment composite			
	N (%)	OR (95%CI)^**1**^		***P*** value
Fruit (≥twice per day)	173 (58.1%)	**0.40 (0.26–0.61)**		**<.001**
Vegetables (≥twice per day)	239 (80.2%)	**0.30 (0.18–0.52)**		**<.001**
Energy-dense snacks (≥once per day)	224 (75.2%)	**1.71 (1.08–2.69)**		**.022**
Fast food intake (≥once per week)	59 (19.8%)	**3.09 (1.90–5.04)**		**<.001**
Convenience food (≥twice per week)	106 (35.6%)	**2.58 (1.64–4.05)**		**<.001**
Sugar Sweetened Beverages (≥once per day)	25 (8.4%)	1.61 (0.92–2.82)		.097
Artificially-sweetened beverages (≥once per day)	97 (32.6%)	**1.54 (1.03–2.29)**		**.034**
Fruit juice (≥once per day)	125 (41.9%)	0.93 (0.66–1.31)		.678
Milk (≥twice per day)	85 (28.6%)	1.36 (0.97–1.93)		.076
**Activity behaviours**	**N (%)**	**OR (95% CI)**		
Physical activity (more active)	177 (59.4%)	**0.57 (0.40–0.80)**		**.002**
**Screen-based sedentary behaviours**^b^	**Mean (SD)**	**Β (**±**SE)**	**R**^**2**^	
TV viewing and screen time (hours/ week)	16.73 (9.70)	**4.55 (**±.78)	**.175**	**<.001**
Video game use (hours/week)	6.91 (6.82)	**1.56 (**±.43)	**.325**	**<.001**

*OR* Odds Ratio, 95% CI = 95% confidence interval

^a^Adjusting for clustering within families (complex samples analyses), the child’s age at time of home environment interview, child sex

^b^Sedentary behaviours were treated as a continuous variable as there are no specific guidelines for duration of screen-time and video game use in this age group [30]

Each 1 unit increase in obesogenic risk in home environments was associated with children being 43% less physically active (OR; 95% CI = 0.57; 0.40–0.80, *p* < .01). Children living in ‘higher-risk’ home environments also had significantly higher overall screen-time (TV viewing and online media content) (*β (SE)* = 4.55 (.78), *p* < .001) and higher video game use (*β (SE)* = 1.56 (.43), *p* < 0.001), such that children’s overall screen-time was 4.55 units (hours/week) higher and video game use was 1.56 units (hours/week) higher for each 1 unit increase in obesogenic risk in the overall home environment.

As shown in Table 5, ‘higher-risk’ overall home environment was associated with higher BMI-SDS at age 12 *(β (SE) = 0.23 (0.09), p = .014),* such that children’s BMI-SDS was 0.23 units higher for each 1 unit increase in obesogenic risk of the overall home environment*.* Additionally, ‘higher-risk’ media environments were associated with higher BMI-SDS at age 12 (*β (SE) = 0.12 (0.03), p < .001*). No association was observed between the activity and food domains and BMI-SDS at 12 years.

**Table 5:** Associations^a^ between the home environment composites and BMI-SDS at age 12 (*N* = 298)

Composite scores	BMI-SDS at age 12		
	β (±SE)	R^**2**^	***P*** value
Home food composite	.006 (±.01)	.002	.674
Home activity composite	−.001 (±.03)	.001	.970
Home Media composite	**.12 (**±**.03)**	**.079**	**<.001**
**Overall home environment composite**	**.23 (**±**.09)**	**.034**	**.014**

^a^Adjusting for clustering within families (complex samples analyses), the child’s age at time of home environment interview, child sex

## Discussion

This study provides evidence in support of the feasibility, reliability and validity of a comprehensive measure of the obesogenic home environment in 12 year old children. The revised HEI was feasible for administration via telephone interviews with primary caregivers. Additionally, the 2-week test-retest reliability of the home environment composite scores were good to excellent. Moreover, this is the first study to demonstrate cross-sectional associations between a comprehensive measure of physical and social aspects of the home environment, and BMI in school-aged children. The findings also characterise relationships between the home environment (overall composite score and the food, activity and media composites separately) and children’s energy balance behaviours (food intake, physical activity and screen-based sedentary behaviours). This reflects similar findings observed in the same cohort when the children were 4 years old [15].

While the observed relationships between the home environment and energy balance behaviours have been previously demonstrated [3, 15, 35, 36], earlier research in this sample found no cross-sectional association with BMI-SDS at age four [15]. However, previous research in this cohort at age four demonstrated that the heritability of BMI was stronger in ‘higher-risk’ home environments compared to ‘lower-risk’ environments [17]. This suggests obesity-related genes are more strongly associated with BMI in more obesogenic environments and children with higher genetic risk for obesity are particularly vulnerable to these obesogenic environments. The positive association between the overall home environment composite score and BMI-SDS observed at age 12 in the present study, supports previous suggestions that the relationship between the home environment and child weight may not manifest until later childhood by which time children have experienced a longer exposure to the home environment, and genetic susceptibility has had the opportunity to be fully expressed in ‘higher-risk’ environments [15, 17]. However, it is important to note that this study is cross-sectional precluding insight into the directionality of these associations. Longitudinal research is needed to examine prospective relationships between the home environment and child weight development.

This finding was also seen for the home media environment with ‘higher-risk’ home media environments being cross-sectionally associated with higher BMI-SDS at 12 years of age. In contrast, the home food and physical activity environment composites were not associated with BMI-SDS in this sample. These findings align with a recent systematic review which highlighted consistent associations between the home media environment and child adiposity [11], but reported mixed findings for the home food and physical activity environments.

The home food environment composite was positively correlated with the home activity *(r = .21, p < .001)* and media composites *(r = .37, p < .001).* This suggests that ‘higher-risk’ in the home food environment was also reflected to some extent in the home activity and media composites*, and* vice versa*.* Conversely, there was no clear association between the home activity and media environments (*r = .03, ns)*, indicating that some aspects of the home may present greater risk for weight gain than others. For example, a household may have a higher score for the media composite, indicating ‘higher-risk’ media environment, but a lower score for the activity composite, indicating ‘lower-risk’ activity environment. This finding is supported by previous research [15] and highlights the importance of utilising measures that capture the overall obesogenic nature of the home environment. Other evidence has suggested that physical activity and sedentary behaviours are largely independent of one another, and engaging in sedentary activities is not necessarily an obstacle to also being physically active [37].

The determinants of physical activity are complex; children’s activity levels are influenced by factors on an individual, interpersonal and environmental level [38, 39]. Existing research has found limited evidence for the role of the home activity environment on children’s physical activity levels [40]. Our findings similarly revealed no association between the home physical activity composite and activity levels in school-aged children. This is perhaps unsurprising, given that as children approach adolescence they increasingly engage in the majority of their physical activity away from the home, through active travel or in school or activity club settings [41–43]. However, associations were observed between the overall home environment and children’s physical activity levels, with children in ‘higher-risk’ home environments found to be less physically active than those living in ‘lower-risk’ home environments. The difference in findings between the overall home environment composite and the individual activity domain highlights the importance of utilising composite measures, as the lower activity levels were largely driven by the home media environment. Other aspects of the home environment likely combine with the activity domain to influence children’s physical activity levels. This view is supported by research conducted in US children (*n* = 713 children, aged 6–11) which found that variables within the home media (e.g. bedroom media devices, parental screen-time) and activity environments (e.g. parental support of PA, PA equipment at home) interact to influence children’s sedentary behaviour and activity levels and, combined, these aspects have greater influence on behaviour than either factor alone [44]. In line with this, the effect sizes for the ORs for the overall home environment composite were substantially larger than for each of individual home environment (food, activity and media) composites, suggesting that the individual aspects of the home environment are correlated with one another, and together have a cumulative effect on childrens’ energy balance behaviours. As such, it is important for future research to utilise composite measures of the home environment, rather than looking at a single domain in isolation.

Evidence highlights that the ‘availability’ and ‘accessibility’ of foods and beverages within the home are important correlates of children’s dietary intake [45–47]. In the present study, children from ‘higher-risk’ home food environments were less likely to consume fruits and vegetables, and were more likely to consume energy-dense snacks, fast food and convenience food. The same patterns of association were observed for the overall home environment composite, but with considerably larger effect sizes. These findings are consistent with previous research conducted in pre-school aged children [15]. However, unlike this previous study [15], findings revealed no association between the home food environment and children’s consumption of sugar-sweetened beverages (SSBs). This difference in findings may be due to the age of the children. At 12 years old, children spend greater time away from the home and have more autonomy over their food choices. It is possible that environments external to the home, are more influential in older children’s consumption of SSBs [10, 48, 49]. However, research conducted in children aged 10–12 years from Eight European countries (*n* = 7915) revealed greater availability of SSB at home was strongly associated with greater consumption of these drinks [50]. Similar findings were also observed in a cross-sectional study of 2719 Australian children aged 11–16 [51]. In both these studies, the children were asked to report their own SSB consumption, whereas the present study utilised parent-reports. Conflicting results may reflect the fact that parents are less aware of their children’s SSB consumption compared to other food and beverage types, or that parents are more susceptible to social desirability biases than their children when reporting dietary intakes [52]. Another possible explanation is that consumption of SSBs was low in the present sample, with only 8.4% of children consuming SSBs ≥once a day, resulting in insufficient variation to observe associations. Future research is required to examine agreement between parent- and child-reported measures of SSB intake in this age group.

In the present research, living in ‘higher-risk’ home media environments (characterised by greater availability of electronic devices in the home and child’s bedroom, fewer parental rules around electronic devices, and greater parental modelling of screen-based sedentary behaviours), was associated with children spending more time watching screens (TV viewing and online media) and playing video games each week. In line with previous research, our findings indicate the environment within the home may be an important factor in shaping children’s behaviours [53]. Therefore, targeting modifiable aspects of the home environment, such as reducing access to electronic devices at home, and setting limits around electronic devices, could be an effective way to reduce screen-time and lower risk for weight gain [10, 54–56]. Furthermore, a recent meta-analysis also revealed that exposure to screen-based junk food advertisements correlates with increases in energy consumption and BMI, these findings suggests that increased exposure to food advertisements via screen time may be another aspect by which the media domain may be predisposing to greater risk for weight gain [57].

Unlike previous research which captures SES using a single indicator (e.g. household income or parental education), the present study utilised a comprehensive composite measure of SES that incorporates individual, household and neighbourhood level factors. In the present study, lower SES was associated with ‘higher-risk’ home environments. These findings are in line with previous research which highlights that lower SES households had greater access to electronic devices in the child’s bedroom [58–60], less access to physical activity equipment and garden space [61–63], and less availability of fruits and vegetables [64–66]. For lower SES families, decisions about food purchasing are largely dictated by price, ease of preparation and a product’s shelf-life. Regular eating routines and family mealtimes are also harder to achieve for caregivers with limited resources and unpredictable working schedules [67, 68]. These factors make it harder for families of lower SES to establish a healthier home environment and must be considered when developing home-based interventions. Future research is needed to examine pathways linking SES, the home environment and weight.

### Strengths and limitations

Strengths of this study include the systematic development and utilisation of a comprehensive measure of the home environment which was guided by expert consultation and cognitive interviews with the target population. This work resulted in a comprehensive measure of the obesogenic home environment that captures composite scores for the food, physical activity and media domains as well as the overall home environment. However, there are several limitations that should be addressed. Firstly, this study relied on parent-report for both the characteristics of the home environment and their children’s energy balance behaviours, and thus may be susceptible to social desirability biases [52]. However, previous research utilising an earlier version of the HEI revealed good to excellent validity when compared to objective measures of the home environment (e.g. wearable devices) [16]. Nevertheless, future research should aim to utilise more objective measures of energy balance behaviours. Although our analyses adjusted for covariates, it is likely that residual confounding from other unmeasured factors remains (i.e. household stress, family dynamics, etc.). It should also be noted that the study sample was small in comparison to the prior study undertaken in participants from the same cohort of children [15]. In comparison, it was also fairly homogenous, with the majority identifying as White (94.6% vs 86.0%) and a large proportion of higher SES households compared with the general population, meaning our findings may not be representative. Furthermore, this study utilised BMI-SDS as the primary measure of adiposity. There are limitations to using BMI as it cannot differentiate between weight attributable to fat mass or lean mass therefore misclassification of weight status can occur at an individual level, especially during later childhood when maturation occurs at differing rates. Thus, utilising other measures of adiposity such as waist circumference, body fat percentage or skinfold thickness may be beneficial. Finally, the cross-sectional nature of this research prevents conclusions regarding the directionality of observed relationships and causality cannot be established. It is possible that children’s energy balance behaviours and/or adiposity influence the home environment, or that the association is bidirectional. Future longitudinal research is required: (1) to examine the stability of the home environment over time, (2) to understand the role of the obesogenic home environment on children’s weight development from early childhood to adolescence, and (3) to investigate the direction of associations between the home environment and adiposity in childhood.

## Conclusion

This study revealed cross-sectional associations between the overall home environment composite score and dietary intake, physical activity and screen-based sedentary behaviours in 12-year-old children. These findings mirror similar observations in the same sample at age four. However, contrary to the earlier findings, positive associations were also observed between BMI-SDS and the overall home environment composite and the home media environment composite. This study provides further evidence for the importance of utilising composite measures of the overall home environment to understand relationships between the home environment and children’s health behaviours and weight trajectories across childhood.

## Supplementary Information

**Additional file 1: Table S1.** Experts’ categorisation of the home food, activity and media environment variables (% (n)). **Table S2.** Constructs included in the home environment composite score. (Items coloured red are those added to the original composite score during the update).

## Data Availability

The datasets used and/or analysed during the current study are available from the corresponding author on reasonable request.

## Abbreviations

HEI: Home Environment Interview
OR: Odds Ratio
PA: Physical Activity
SSB: Sugar Sweetened beverages
BMI: Body Mass Index
SDS: Standard Deviation Score
SES: Socioeconomic Status

## References

[1] Couch SC, Glanz K, Zhou C, Sallis JF, Saelens BE (2014). Home food environment in relation to children’s dietquality and weight status. J Acad Nutr Diet.

[2] Rosenkranz RR, Dzewaltowski DA (2008). Model of the home food environment pertaining to childhood obesity. Nutr Rev.

[3] Hales D, Vaughn AE, Mazzucca S, Bryant MJ, Tabak RG, McWilliams C (2013). Development of HomeSTEAD’s physical activity and screen time physical environment inventory. Int J Behav Nutr Phys Act.

[4] Jago R, Thompson JL, Sebire SJ, Wood L, Pool L, Zahra J (2014). Cross-sectional associations between the screen-time of parents and young children: differences by parent and child gender and day of the week. Int J Behav Nutr Phys Act.

[5] Jago R, Page A, Froberg K, Sardinha LB, Klasson-Heggebø L, Andersen LB (2008). Screen-viewing and the home TV environment: the European youth heart study. Prev Med (Baltim).

[6] Gattshall ML, Shoup J, Marshall JA, Crane LA, Estabrooks PA (2008). Validation of a survey instrument to assess home environments for physical activity and healthy eating in overweight children. Int J Behav Nutr Phys Act.

[7] Pinard CA, Yaroch AL, Hart MH, Serrano EL, McFerren MM, Estabrooks PA (2014). The Validity and reliability of the Comprehensive Home Environment Survey (CHES). Health Promot Pract.

[8] Rosenkranz RR, Dzewaltowski DA (2008). Model of the home food environment pertaining to childhood obesity. Nutr Rev.

[9] Vaughn AE, Hales DP, Neshteruk CD, Ward DS. HomeSTEAD’s physical activity and screen media practices and beliefs survey: Instrument development and integrated conceptual model. Plos One. 2019;14(12) [Cited 2020 Nov 2]. Available from: https://pubmed.ncbi.nlm.nih.gov/31891610/. 10.1371/journal.pone.0226984 PMC6938346 31891610

[10] Verloigne M, Van Lippevelde W, Maes L, Brug J, De Bourdeaudhuij I (2012). Family- and school-based correlates of energy balance-related behaviours in 10–12-year-old children: a systematic review within the ENERGY (EuropeaN Energy balance Research to prevent excessive weight Gain among Youth) project.

[11] Kininmonth A, Smith AD, Llewellyn CH, Dye L, Lawton CL, Fildes A (2021). The relationship between the home environment and child adiposity: a systematic review. Int J Behav Nutr Phys Act.

[12] Golan M (2006). Parents as agents of change in childhood obesity - From research to practice. Int J Pediatr Obes.

[13] Davison KK, Birch LL (2001). Childhood overweight: a contextual model and recommendations for future research. Obes Rev.

[14] Pinard CA, Yaroch AL, Hart MH, Serrano EL, McFerren MM, Estabrooks PA (2012). Measures of the home environment related to childhood obesity: a systematic review. Public Health Nutr.

[15] Schrempft S, Van Jaarsveld CHM, Fisher A, Wardle J (2015). The obesogenic quality of the home environment: Associations with diet, physical activity, TV viewing, and BMI in preschool children. Plos One.

[16] Schrempft S, van Jaarsveld CH, Fisher A (2017). Exploring the potential of a wearable camera to examine the early obesogenic home environment: comparison of sensecam images to the home environment interview. J Med Internet Res.

[17] Schrempft S, van Jaarsveld CM, Fisher A (2018). Variation in the heritability of child body mass index by obesogenic home environment. JAMA Pediatr.

[18] Ofcom (2020). Children and parents: media use and attitudes report 2019.

[19] Ofcom (2019). Children and parents media use and attitudes: annex 1.

[20] Okoli C, Pawlowski SD (2004). The Delphi method as a research tool: an example, design considerations and applications. Inf Manag.

[21] Harris P, Taylor R, Thielke R, Payne J, Gonzalez N, Conde J (2009). Research electronic data capture (REDCap)--a metadata-driven methodology and workflow process for providing translational research informatics support. J Biomed Inform.

[22] Harris PA, Taylor R, Minor B, Elliott V, Fernandez M, O’Neal L, et al. The REDCap consortium: building an international community of software platform partners. J Biomed Inform. 2019;95 [Cited 2021 Aug 13]. Available from: https://pubmed.ncbi.nlm.nih.gov/31078660/. 10.1016/j.jbi.2019.103208 PMC7254481 31078660

[23] Wardle J, Sanderson S, Guthrie CA, Rapoport L, Plomin R (2002). Parental feeding style and the intergenerational transmission of obesity risk. Obes Res.

[24] Birch LL, Fisher JO, Grimm-Thomas K, Markey CN, Sawyer R, Johnson SL (2001). Confirmatory factor analysis of the child feeding questionnaire: a measure of parental attitudes, beliefs and practices about child feeding and obesity proneness. Appetite.

[25] Musher-Eizenman D, Holub S (2007). Comprehensive feeding practices questionnaire: Validation of a new measure of parental feeding practices. J Pediatr Psychol.

[26] Ogden J, Reynolds R, Smith A (2006). Expanding the concept of parental control: a role for overt and covert control in children’s snacking behaviour?. Appetite.

[27] van Jaarsveld CH, Johnson L, Llewellyn C, Gemini WJ (2010). A UK twin birth cohort with a focus on early childhood weight trajectories, appetite and the family environment. Twin Res Hum Genet.

[28] Roe L, Strong C, Whiteside C, Neil A, Mant D (1994). Dietary intervention in primary care: Validity of the dine method for diet assessment. Fam Pract..

[29] Purslow LR, van Jaarsveld CHM, Semmler C, Wardle J (2009). Validity and prognostic value of parental ratings of children’s activity. Prev Med (Baltim)..

[30] Hill D, Ameenuddin N, Chassiakos YR, Cross C, Radesky J, Hutchinson J, et al. Media use in school-aged children and adolescents. Pediatrics. 2016;138(5) [Cited 2021 Feb 4]. Available from: www.aappublications.org/news. 10.1542/peds.2016-2592 27940794

[31] Kininmonth AR, Smith AD, Llewellyn CH, Fildes A (2019). Socioeconomic status and changes in appetite from toddlerhood to early childhood. Appetite.

[32] Freeman JV, Cole TJ, Chinn S, Jones PR, White EM, Preece MA (1995). Cross sectional stature and weight reference curves for the UK, 1990. Arch Dis Child.

[33] IBM Corp (2019). IBM SPSS statistics for windows, version 26.0.

[34] Fleiss J (1986). Reliability of measurement. The design and analysis of clinical experiments.

[35] Verloigne M, Van LW, Maes L, Brug J, De BI (2012). Family- and school-based correlates of energy balance-related behaviours in 10–12-year-old children: a systematic review within the ENERGY (EuropeaN Energy balance Research to prevent excessive weight Gain among Youth) project. Public Health Nutr.

[36] Pinard CA, Yaroch AL, Hart MH, Serrano EL, MM MF, Estabrooks PA (2014). The validity and reliability of the Comprehensive Home Environment Survey (CHES). Health Promot Pract.

[37] Biddle SJH, Gorely T, Marshall SJ, Murdey I, Cameron N (2004). Physical activity and sedentary behaviours in youth: issues and controversies. J R Soc Promot Health.

[38] SJH B, Atkin AJ, Cavill N, Foster C (2011). Correlates of physical activity in youth: a review of quantitative systematic reviews. Int Rev Sport Exerc Psychol.

[39] Wilk P, Clark AF, Maltby A, Smith C, Tucker P, Gilliland JA (2018). Examining individual, interpersonal, and environmental influences on children’s physical activity levels. SSM - Popul Heal.

[40] Maitland C, Stratton G, Foster S, Braham R, Rosenberg M (2013). A place for play? The influence of the home physical environment on children’s physical activity and sedentary behaviour. Int J Behav Nutr Phys Act.

[41] Rainham DG, Bates CJ, Blanchard CM, Dummer TJ, Kirk SF, Shearer CL (2012). Spatial classification of youth physical activity patterns. Am J Prev Med.

[42] Dunton GF, Kawabata K, Intille S, Wolch J, Pentz MA (2012). Assessing the social and physical contexts of children’s leisure-time physical activity: an ecological momentary assessment study. Am J Heal Promot.

[43] Engelen L, Bundy AC, Lau J, Naughton G, Wyver S, Bauman A (2015). Understanding patterns of young children’s physical activity after school - It’s all about context: a cross-sectional study. J Phys Act Heal.

[44] Tandon P, Grow HM, Couch S, Glanz K, Sallis JF, Frank LD (2014). Physical and social home environment in relation to children’s overall and home-based physical activity and sedentary time. Prev Med (Baltim).

[45] Cook LT, O’Reilly GA, Derosa CJ, Rohrbach LA, Spruijt-Metz D (2015). Association between home availability and vegetable consumption in youth: a review.

[46] Dave JM, Evans AE, Pfeiffer KA, Watkins KW, Saunders RP (2010). Correlates of availability and accessibility of fruits and vegetables in homes of low-income Hispanic families. Health Educ Res.

[47] Hanson NI, Neumark-Sztainer D, Eisenberg ME, Story M, Wall M (2005). Associations between parental report of the home food environment and adolescent intakes of fruits, vegetables and dairy foods. Public Health Nutr.

[48] Hebden L, Hector D, Hardy LL, King L (2013). A fizzy environment: availability and consumption of sugar-sweetened beverages among school students. Prev Med (Baltim).

[49] Bere E, Sørli Glomnes E, Te Velde SJ, Klepp KI (2008). Determinants of adolescents’ soft drink consumption. Public Health Nutr.

[50] Van Lippevelde W, te Velde SJ, Verloigne M, De Bourdeaudhuij I, Manios Y, Bere E (2013). Associations between home- and family-related factors and fruit juice and soft drink intake among 10- to 12-year old children. The ENERGY project. Appetite..

[51] Denney-Wilson E, Crawford D, Dobbins T, Hardy L, Okely DEdAD (2009). Influences on consumption of soft drinks and fast foods in adolescents. Asia Pac J Clin Nutr..

[52] Bornstein MH, Putnick DL, Lansford JE, Pastorelli C, Skinner AT, Sorbring E (2015). Mother and father socially desirable responding in nine countries: two kinds of agreement and relations to parenting self-reports. Int J Psychol.

[53] Granich J, Rosenberg M, Knuiman MW, Timperio A (2011). Individual, social, and physical environment factors associated with electronic media use among children: Sedentary behavior at home. J Phys Act Heal.

[54] Bjelland M, Soenens B, Bere E, Kovács É, Lien N, Maes L (2015). Associations between parental rules, style of communication and children’s screen time Health behavior, health promotion and society. BMC Public Health.

[55] Ramirez ER, Norman GJ, Rosenberg DE, Kerr J, Saelens BE, Durant N (2011). Adolescent screen time and rules to limit screen time in the home. J Adolesc Heal.

[56] Saelens BE, Sallis JF, Nader PR, Broyles SL, Berry CC, Taras HL (2002). Home environmental influences on children’s television watching from early to middle childhood. J Dev Behav Pediatr.

[57] Russell SJ, Croker H, Viner RM (2019). The effect of screen advertising on children’s dietary intake: A systematic review and meta-analysis. Obes Rev.

[58] Mihrshahi S, Drayton BA, Bauman AE, Hardy LL (2017). Associations between childhood overweight, obesity, abdominal obesity and obesogenic behaviors and practices in Australian homes. BMC Public Health.

[59] Adachi-Mejia A, Longacre M, Gibson J, Beach M, Titus-Ernstoff L, Dalton M (2007). Children with a TV in their bedroom at higher risk for being overweight. Int J Obes.

[60] Borghese MM, Tremblay MS, Katzmarzyk PT, Tudor-Locke C, Schuna JM, Leduc G (2015). Mediating role of television time, diet patterns, physical activity and sleep duration in the association between television in the bedroom and adiposity in 10 year-old children. Int J Behav Nutr Phys Act.

[61] Schalkwijk AA, van der Zwaard BC, Nijpels G, Elders PJ, Platt L (2018). The impact of greenspace and condition of the neighbourhood on child overweight. Eur J Public Health.

[62] Tandon P, Zhou C, Sallis JF, Cain KL, Frank LD, Saelens BE (2012). Home environment relationships with children’s physical activity, sedentary time, and screen time by socioeconomic status. Int J Behav Nutr Phys Act.

[63] Umstattd Meyer MR, Sharkey JR, Patterson MS, Dean WR (2013). Understanding contextual barriers, supports, and opportunities for physical activity among Mexican-origin children in Texas border colonias: a descriptive study. BMC Public Health.

[64] Boles RE, Johnson SL, Burdell A, Davies PL, Gavin WJ, Bellows LL (2019). Home food availability and child intake among rural families identified to be at-risk for health disparities. Appetite.

[65] Carolyn Grunseit A, Taylor AJ, Lawson Hardy L, King L. Composite measures quantify households’ obesogenic potential and adolescents’ risk behaviors128:e000. Pediatrics. 2011;128(2) [Cited 2021 Jan 18]. Available from: www.pediatrics.org/cgi/doi/10.1542/peds.2010-3331. 10.1542/peds.2010-3331 21727105

[66] Schrempft S, van Jaarsveld CHM, Fisher A, Fildes A, Wardle J (2016). Maternal characteristics associated with the obesogenic quality of the home environment in early childhood. Appetite.

[67] Bauer KW, Hearst MO, Escoto K, Berge JM, Neumark-Sztainer D (2012). Parental employment and work-family stress: associations with family food environments. Soc Sci Med.

[68] Shift. Families and Food: how the environment influences what families eat. 2018. Available from: https://www.gsttcharity.org.uk/sites/default/files/Bite_Size_Report.pdf

