# Supplementary material for: The Home Environment Interview and associations with energy balance behaviours and body weight in school-aged children – a feasibility, reliability, and validity study
Source: Int J Behav Nutr Phys Act. 2021 Dec 23;18:167. doi: 10.1186/s12966-021-01235-3 (PMC8698661; doi:10.1186/s12966-021-01235-3)
Supplement: Supplementary file 1 — Additional file 1: Table S1. Experts’ categorisation of the home food, activity and media environment variables (% (n)). Table S2. Constructs included in the home environment composite score. (Items coloured red are those added to the original composite score during the update). [file 12966_2021_1235_MOESM1_ESM.docx]

**Supplementary materials**

**Table S1.** Experts’ categorisation of the home food, activity and media environment variables (% (n)).

|  | **Probably/definitely INCREASED risk** | **Probably/definitely DECREASED risk** | **Not sure** |
| --- | --- | --- | --- |
| **Home Food Environment Variables** | | | |
| More types of fruit in the home | 0.0 (0) | **80.9 (17)** | 19.1 (4) |
| Fruit on display (visible) | 0.0 (0) | **85.7 (18)** | 14.3 (3) |
| Child is allowed to help themselves to fruit | 0.0 (0) | **71.4 (15)** | 28.6 (6) |
| More types of vegetables in the home | 0.0 (0) | **90.5 (19)** | 9.5 (2) |
| Ready-to-eat vegetables in the fridge or on the kitchen counter | 0.0 (0) | **85.7 (18)** | 14.3 (3) |
| Child allowed to help themselves to vegetables | 0.0 (0) | **76.2 (16)** | 23.8 (5) |
| More types of energy-dense snack in the home | **90.5 (19)** | 0.0 (0) | 9.5 (2) |
| Child allowed to help themselves to energy-dense snack | **95.3 (20)** | 0.0 (0) | 4.8 (1) |
| Energy-dense snacks on display (visible) | **90.5 (19)** | 0.0 (0) | 9.5 (2) |
| Fruit juice or smoothies in the home | 33.3 (7) | 0.0 (0) | 66.7 (14) |
| Fruit juice or smoothies on display (visible) | 38.1 (8) | 0.0 (0) | 61.9 (13) |
| Full-fat milk in the home | 19.1 (4) | 14.3 (3) | 66.7 (14) |
| Skimmed milk in the home | 0.0 (0) | 38.1 (8) | 61.9 (13) |
| Semi-skimmed milk in the home | 0.0 (0) | 33.3 (7) | 66.7 (14) |
| Child is allowed to help themselves to milk | 14.3 (3) | 23.8 (5) | 61.9 (13) |
| Child is allowed to help themselves to fruit juice or smoothies | 57.1 (12) | 0.0 (0) | 42.86 (9) |
| Sugar-sweetened drinks in the home | **95.2 (20)** | 0.0 (0) | 4.76 (1) |
| Sugar-sweetened drinks on display (visible) | **90.5 (19)** | 0.0 (0) | 9.52 (2) |
| Child is allowed to help themselves to sugar-sweetened drinks | **100.0 (21)** | 0.0 (0) | 0.00 (0) |
| Family meals at the table | 0.0 (0) | **90.48 (19)** | 9.52 (2) |
| Sugar-free drinks in the home (excluding water) | 9.5 (2) | 28.57 (6) | 61.90 (13) |
| Sugar-free drinks (excluding water) on display (visible) | 9.5 (2) | 28.57 (6) | 61.90 (13) |
| Child is allowed to help themselves to sugar-free drinks (excluding water) | 9.5 (2) | 23.81 (5) | 66.67(14) |
| Caffeinated energy drinks in the home | 45.0 (9) | 0.00 (0) | 55.00 (11) |
| Caffeinated energy drinks on display (visible) | 42.9 (9) | 0.00 (0) | 57.14 (12) |
| Child is allowed to help themselves to caffeinated energy drinks in the home | 52.4 (11) | 0.00 (0) | 47.62 (10) |
| Greater frequency of meals eaten together at the table as a family | 0.0 (0) | **90.48 (19)** | 9.52 (2) |
| Parental rules around family mealtimes | 0.0 (0) | **71.43 (15)** | 28.57 (6) |
| Parental restriction of unhealthy foods | 4.8 (1) | **76.19 (16)** | 19.05 (4) |
| Parental use of food to make child feel better | **76.2 (16)** | 4.76 (1) | 19.05 (4) |
| Parental use of food as a reward | **90.5 (19)** | 0.00 (0) | 9.52 (2) |
| Parental encouragement for the child to eat fruit and vegetables | 0.0 (0) | **85.00 (17)** | 15.00 (3) |
| Parental monitoring of the child's unhealthy food intake | 0.0 (0) | **85.71 (18)** | 14.29 (3) |
| Parental control of child's food intake | 28.6 (6) | 38.10 (8) | 33.33 (7) |
| Parental pressure for the child to eat | **71.4 (15)** | 0.00 (0) | 28.57 (6) |
| Parental covert restriction of child's unhealthy food intake | 0.0 (0) | **66.67 (14)** | 33.33 (7) |
| **Home Media Environment Variables** | | | |
| Greater amount of media equipment in child's bedroom (e.g. TV, computer, games consoles, laptops, tablets, phones) | **95.2 (20)** | 0.0 (0) | 4.8 (1) |
| Greater amount of media equipment in the home (e.g. TVs, DVD players, games consoles, laptops, tablets, mobile phones) | **76.2 (16)** | 0.0 (0) | 23.8 (5) |
| Greater maternal time engaged in screen-based viewing | **76.2 (16)** | 0.0 (0) | 23.8 (5) |
| Greater paternal time engaged in screen-based viewing | **76.2 (16)** | 0.0 (0) | 23.8 (5) |
| Parental rules around use of media equipment | 4.8 (1) | **76.2 (16)** | 19.0 (4) |
| Greater maternal time playing video games | 57.1 (12) | 0.0 (0) | 42.9 (9) |
| Greater paternal time playing video games | 57.1 (12) | 0.0 (0) | 42.9 (9) |
| Parental use of electronic device time (e.g. phone, TV, computer, games console) as a reward | 42.9 (9) | 9.5 | 47.6 (10) |
| Parental limits on media equipment time (e.g. phone, TV, computer) if the child misbehaves | 0.0 (0) | 28.6 | 71.4 (15) |
| Child eats whilst watching TV or using an electronic device | **85.0 (17)** | 5.0 | 10.0 (2) |
| Greater frequency of eating whilst watching TV or using an electronic device | **80.9 (17)** | 4.8 | 14.3 (3) |
| Child has access to and use of social media | 33.3 (7) | 9.5 (2) | 57.1 (12) |
| Parental rules around child's online activities and social media use | 0.0 (0) | 57.1 (12) | 42.9 (9) |
| **Home Physical Activity Variables** | | | |
| Greater frequency that the child is allowed to be physically active in the garden/yard. | 0.0 (0) | **95.2 (20)** | 4.8 (1) |
| Greater frequency that the child is allowed to be physically active inside the home. | 0.0 (0) | **80.9 (17)** | 19.1 (4) |
| Play equipment in the garden/yard. | 0.0 (0) | **85.7 (17)** | 14.3 (3) |
| Garden/yard that the child can play in | 0.0 (0) | **80.9 (18)** | 19.1 (4) |
| Outdoor recreation area close to home | 0.0 (0) | 57.1 (12) | 42.9 (9) |
| Larger garden/yard that the child can be active in vs smaller garden/yard | 0.0 (0) | 52.4 (11) | 47.6 (10) |
| Indoor recreation centres close to home | 0.0 (0) | 45.0 (9) | 55.0 (11) |
| Child has a usable bike, scooter, rollerblades or skateboard. | 0.0 (0) | **61.9 (13)** | 38.1 (8) |
| Parental modelling of physical activity | 0.0 (0) | **100.0 (21)** | 0.0 (0) |
| Parental support of physical activity | 0.0 (0) | **100.0 (20)** | 0.0 (0) |

| **Version 1: HE Composite 2012** | **Version 2: Updated HE Composite** |
| --- | --- |
| **Food-related constructs (21)** | **Food-related constructs (21)** |
| ***Availability*** | ***Availability*** |
| Number of fruit types[^1^](https://www.sciencedirect.com/science/article/pii/S0195666316304251#tbl1fna) | Number of fruit types[^1^](https://www.sciencedirect.com/science/article/pii/S0195666316304251#tbl1fna) |
| Number of vegetable types[^1^](https://www.sciencedirect.com/science/article/pii/S0195666316304251#tbl1fna) | Number of vegetable types[^1^](https://www.sciencedirect.com/science/article/pii/S0195666316304251#tbl1fna) |
| Number of energy-dense snack types | Number of energy-dense snack types |
| Presence of sugar-sweetened drinks | Number of sugar-sweetened drinks |
| ***Accessibility (visibility)*** | ***Accessibility (visibility)*** |
| Fruit on display[^1^](https://www.sciencedirect.com/science/article/pii/S0195666316304251#tbl1fna) | Fruit on display[^1^](https://www.sciencedirect.com/science/article/pii/S0195666316304251#tbl1fna) |
| Vegetables ready-to-eat[^1^](https://www.sciencedirect.com/science/article/pii/S0195666316304251#tbl1fna) | Vegetables ready-to-eat[^1^](https://www.sciencedirect.com/science/article/pii/S0195666316304251#tbl1fna) |
| Energy-dense snacks on display | Energy-dense snacks on display |
| Sugar-sweetened drinks on display | Sugar-sweetened drinks on display |
| ***Accessibility (child can help him/herself)*** | ***Accessibility (child can help him/herself)*** |
| Fruit[^1^](https://www.sciencedirect.com/science/article/pii/S0195666316304251#tbl1fna) | Fruit[^1^](https://www.sciencedirect.com/science/article/pii/S0195666316304251#tbl1fna) |
| Vegetables[^1^](https://www.sciencedirect.com/science/article/pii/S0195666316304251#tbl1fna) | Vegetables[^1^](https://www.sciencedirect.com/science/article/pii/S0195666316304251#tbl1fna) |
| Energy-dense snacks | Energy-dense snacks |
| Sugar-sweetened drinks | Sugar-sweetened drinks |
| ***Parental feeding practices*** | ***Parental feeding practices*** |
| Emotional feeding | Emotional feeding |
| Instrumental feeding | Instrumental feeding |
| Encouragement[^1^](https://www.sciencedirect.com/science/article/pii/S0195666316304251#tbl1fna) | Encouragement[^1^](https://www.sciencedirect.com/science/article/pii/S0195666316304251#tbl1fna) |
| Modelling[^1^](https://www.sciencedirect.com/science/article/pii/S0195666316304251#tbl1fna) | Modelling[^1^](https://www.sciencedirect.com/science/article/pii/S0195666316304251#tbl1fna) |
| Monitoring[^1^](https://www.sciencedirect.com/science/article/pii/S0195666316304251#tbl1fna) | Monitoring[^1^](https://www.sciencedirect.com/science/article/pii/S0195666316304251#tbl1fna) |
| Covert restriction[^1^](https://www.sciencedirect.com/science/article/pii/S0195666316304251#tbl1fna) | Covert restriction[^1^](https://www.sciencedirect.com/science/article/pii/S0195666316304251#tbl1fna) |
| Restriction[^1^](https://www.sciencedirect.com/science/article/pii/S0195666316304251#tbl1fna) | Restriction[^1^](https://www.sciencedirect.com/science/article/pii/S0195666316304251#tbl1fna) |
| Family meal frequency at the table | Family meal frequency at the table |
| Frequency child eats while watching TV | Frequency child eats while watching TV and/or using a device |
| **Physical activity-related constructs (6)** | **Physical activity-related constructs (6)** |
| Garden/outdoor space[^1^](https://www.sciencedirect.com/science/article/pii/S0195666316304251#tbl1fna) | Garden/outdoor space[^1^](https://www.sciencedirect.com/science/article/pii/S0195666316304251#tbl1fna) |
| Garden play equipment[^1^](https://www.sciencedirect.com/science/article/pii/S0195666316304251#tbl1fna) | Garden play equipment[^1^](https://www.sciencedirect.com/science/article/pii/S0195666316304251#tbl1fna) |
| Allowed to play indoors[^1^](https://www.sciencedirect.com/science/article/pii/S0195666316304251#tbl1fna) | Allowed to play indoors[^1^](https://www.sciencedirect.com/science/article/pii/S0195666316304251#tbl1fna) |
| Allowed to play outdoors[^1^](https://www.sciencedirect.com/science/article/pii/S0195666316304251#tbl1fna) | Allowed to play outdoors[^1^](https://www.sciencedirect.com/science/article/pii/S0195666316304251#tbl1fna) |
| Caregiver modelling of physical activity[^1^](https://www.sciencedirect.com/science/article/pii/S0195666316304251#tbl1fna) | Caregiver modelling of physical activity[^1^](https://www.sciencedirect.com/science/article/pii/S0195666316304251#tbl1fna) |
| Caregiver support of physical activity[^1^](https://www.sciencedirect.com/science/article/pii/S0195666316304251#tbl1fna) | Caregiver support of physical activity[^1^](https://www.sciencedirect.com/science/article/pii/S0195666316304251#tbl1fna) |
| **Media-related constructs (5)** | **Media-related constructs (5)** |
| Number of media equipment in home | Number of media equipment in home |
| TV in the child’s bedroom | Number of media equipment in child’s bedroom |
| Household rules around media use[^1^](https://www.sciencedirect.com/science/article/pii/S0195666316304251#tbl1fna) | Caregiver rules around use of media equipment[^1^](https://www.sciencedirect.com/science/article/pii/S0195666316304251#tbl1fna) |
| TV viewing of primary caregiver (hrs. per week) | Primary caregiver time engaged in screen-based viewing (hrs. per week) |
| TV viewing of partner (hrs. per week) | Partner time engaged in screen-based viewing (hrs. per week) |
| ^1^ Variable was identified as being associated with decreased risk for weight gain. | |

**Table S2.** Constructs included in the home environment composite score. (Items coloured red are those added to the original composite score during the update).
